# Significance of Worst Pattern of Invasion-5 in Early-Stage Oral Cavity Squamous Cell Carcinoma

**DOI:** 10.1007/s12105-023-01571-9

**Published:** 2023-07-24

**Authors:** Shima Mohamed, Hadeel Jawad, Ryan O’ Sullivan, Deirdre Callanan, Patrick Sheahan, Linda Feeley

**Affiliations:** 1 Department of Pathology, Cork University Hospital, Wilton, Cork, T12 DC4A Ireland; 2 Department of Otolaryngology, South Infirmary Victoria University Hospital, Cork, Ireland; 3 Department of Surgery, University College, Cork, Ireland; 4 ENTO Research Unit, College of Medicine and Health, University College Cork, Cork, Ireland; 5 Present Address: St James’s Hospital, Dublin, Ireland; 6 Present Address: Black Country Pathology Services, NHS, Wolverhampton, UK

**Keywords:** Oral cancer, Head and neck cancer, Squamous cell carcinoma, Prognosis, Worst pattern of invasion

## Abstract

**Background:**

There is an ongoing need to identify pathologic prognosticators in early-stage oral cavity squamous cell carcinoma (OCSCC) to aid selection of patients who may benefit from adjuvant treatment. The objective of this study was to evaluate the prognostic ability of worst pattern of invasion-5 (WPOI-5) defined by the presence of satellite nodules, extratumoural perineural invasion (PNI) and/or extratumoural lymphovascular space invasion (LVI) in low-stage, node negative OCSCC.

**Methods:**

This was a retrospective study of 160 patients with T1/T2N0 tumours staged using TNM7 treated surgically. Histology of the primary tumour was re-reviewed as appropriate to assess for the presence of WPOI-5 parameters. Univariate and multivariate analysis assessing impact of pathological features on survival outcomes was performed.

**Results:**

On univariate analysis, WPOI-5 and its 3 constituent components of satellite nodules, extratumoural PNI and extratumoural LVI were all significantly associated with disease-specific survival (DSS) and overall survival (OS). On multivariate analysis, satellite nodules (odds ratio 6.61, 95% CI 2.83–15.44, *p* < 0.0001) and extratumoural LVI (odds ratio 9.97, 95% CI 2.19–45.35, *p* = 0.003) were independently associated with OS. Postoperative radiotherapy (odds ratio 0.40, 95% CI 0.19–0.87, *p* = 0.02) and non-tongue subsite (odds ratio 3.03, 95% CI 1.70–5.39, *p* = 0.0002) were also significantly associated with OS on multivariate analysis.

**Conclusion:**

Satellite nodules and extratumoural LVI correlated significantly with survival outcomes in our early-stage OSCC cohort. Further study is required to investigate the benefit of adjuvant treatment in these cases and to ascertain if WPOI-5 parameters including satellite nodules should be mandatory reporting data elements.

## Introduction

Tumour stage at diagnosis is an important but imperfect prognosticator in oral cavity squamous cell carcinoma (OCSCC) [1]. A significant proportion of patients with small (T1/T2), node negative primaries categorised using tumour, nodes, metastasis (TNM) classification 7th edition (TNM7) went on to develop local recurrence and/or nodal metastatic spread with reported disease-specific mortality rates as high as 25% and 37% for stage I and II disease, respectively [2]. In an attempt to improve the performance of the oral cavity cancer staging system, the American Joint Committee on Cancer Control (AJCC) incorporated depth of invasion (DOI) into the T staging of the updated TNM8 2017 [3]. Multiple studies have demonstrated DOI to significantly predict outcome in OCSCC [4–11] with 5 mm and 10 mm selected as the cut points for tumour upstaging based on a retrospective analysis undertaken by the International Consortium for Outcome Research in Head and Neck Cancer (ICOR) of 3149 patients treated at 11 cancer centres between 1990 and 2011 [11].

Even with the adoption of TNM8 there is an ongoing need to identify further robust pathologic prognosticators in early-stage OCSCC to aid selection of patients who may benefit from adjuvant radiotherapy. Margin status is proven to significantly predict local recurrence [12–16]. Other parameters that have been shown to correlate with recurrence and survival outcomes in OCSCC include perineural invasion (PNI) [17–25], lymphovascular space invasion (LVI) [22, 24, 26–31] and pattern of invasion (POI) at the invasive tumour front [19, 21, 32–34]. PNI and LVI are regarded by both the Royal College of Pathologists (RCPath) and College of American Pathologists (CAP) to be core data elements for reporting [35, 36]. In relation to POI, the CAP designates worst pattern of invasion (WPOI) as non-core and optional for reporting. Although 5 patterns of invasion are recognized, distinction of WPOI-5 (defined as dispersion of ≥ 1 mm between tumour satellites), from patterns 1 to 4 is most significant [19]. Of note, extratumoural PNI and extratumoural LVI also qualify as WPOI-5 [36]. In contrast the RCPath classifies POI using a two-tier system as cohesive versus non-cohesive and lists it as a required data item. Of note, non-cohesive invasive front includes both patterns 4 and 5, and a quantitative cut-off (i.e., worst pattern or other) is not explicitly specified in the RCPath dataset currently [35].

The primary aim of this study was to evaluate the prognostic ability of WPOI-5 including extratumoural PNI and extratumoural LVI in early-stage oral cavity squamous cell carcinoma (OCSCC). The patient cohort comprised T1/T2N0 tumours staged using TNM7.

## Materials and Methods

The study comprised a retrospective review of 160 patients with OCSCC who underwent definitive primary surgical treatment at the South Infirmary Victoria University Hospital, Cork, Ireland between the years 2000 and 2020. Ethical approval was granted by the Cork Clinical Research Ethics Committee. Inclusion criteria were patients with pT1/2N0 (TNM7) cancers undergoing primary surgical treatment. Exclusion criteria were patients with cervical or distant metastases; previous history of head and neck cancer or treatment with radiotherapy; or with synchronous primary head and neck cancers. Patients not undergoing neck dissection were included provided there was no clinical or radiological evidence of cervical metastases (cN0). Cases were identified and relevant clinical and pathological data extracted from our cancer database having been previously populated from review of medical charts, histological reports and/or slide review.

Clinical data recorded included sex, site of cancer within the oral cavity, smoking status, alcohol use, administration of post-operative radiotherapy, and clinical outcome including recurrence, death due to cancer, death due to other causes, and further primary cancers. Pathological data recorded included depth of invasion, TNM8 T-stage, and margin status. Of note, depth of invasion (DOI) measurements were previously re-evaluated for all patients in our study cohort who underwent surgery prior to the issuance by the AJCC in 2017 of the clarified definition for this parameter [3]. Margin positivity was classified using both the RCPath definition of invasive carcinoma within 1 mm of margin [35] and the CAP definition of invasive carcinoma or high-grade dysplasia present at margin [36]. Margin status was based in all cases on the main specimen, and not taking into consideration any separately submitted frozen sections or extra tumour bed resections. In addition, haematoxylin and eosin (H&E)-stained slides of the primary tumour were re-reviewed in (1) cases reported as having a non-cohesive invasive front, to ascertain if satellite nodules fulfilling the definition for WPOI-5 were present; (2) all cases with PNI, to classify the location as intratumoural and/or extratumoural; and (3) all cases with LVI, to classify the location as intratumoural and/or extratumoural.

**Fig. 1:**
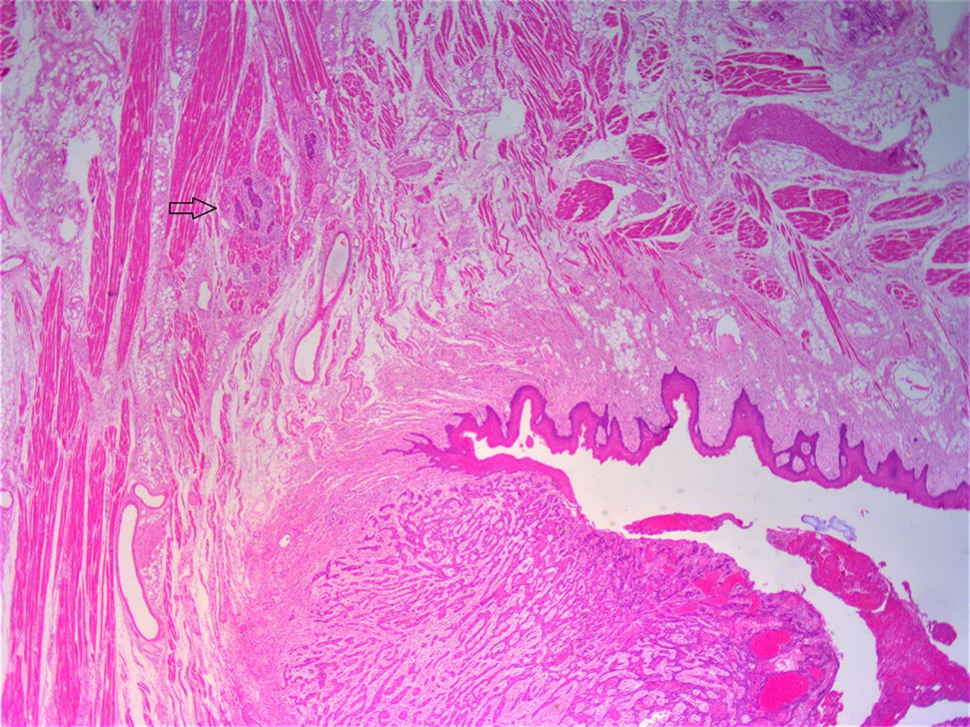
Low power (1.5×) H&E image showing main tumour bottom right and a satellite focus > 1 mm away at the black arrow

Slide review was undertaken by 2 pathologists at a multiheaded microscope and consensus results recorded. In cases with PNI and LVI reported, this was re-confirmed by the 2 reviewing pathologists by agreement. There was some re-categorisation of cases from PNI/LVI positive to negative and vice versa, which was expected due to interobserver variability. Extratumoural PNI and extratumoural LVI were defined as PNI or LVI present ≥ 1 mm beyond the invasive tumour front, respectively. Cases not fulfilling the criterion for extratumoural in location were classified as intratumoural. WPOI-5 was defined as dispersed satellite nodule(s) ≥ 1 mm from the main tumour or next closest satellite (Fig. 1). Satellite nodules were distinguished from regional soft tissue deposits in level IA based on location (present in tumour sections and in close proximity to, but sufficiently beyond the invasive tumour front i.e. at least 1 mm); size (present as small clusters); shape (irregular, non-rounded); and lack of associated lymphoid tissue. In addition, extratumoural PNI and extratumoural LVI were also classified as WPOI-5. Therefore, 3 categories of WPOI-5 were recognized, which were evaluated both separately and collectively when statistical analysis was performed.

XLSTAT (Addinsoft, France, version 2015.1.03) was utilised for statistical analysis. Survival was calculated from time of surgery to time of death or last clinic follow-up. Patients dying with recurrence or uncontrolled cancer were regarded to have died from disease. Patients dying from medical complications in the first month after surgery were also classified as having died due to cancer [37, 38]. Locoregional recurrence (LRR)-free survival was calculated from time of surgery to time of diagnosis of local or regional recurrence, or to last clinical follow-up. Local recurrence was considered to be present in any patient with recurrent cancer at the same or contiguous subsite of the oral cavity as the originally presenting cancer, regardless of interval since the original surgery. Patients presenting with true second primary head and neck cancers were censored on the date or presentation with the second primary. Survival estimates were performed using the Kaplan–Meier method. Univariate and multivariate analysis assessing impact of pathological features on survival outcomes was performed using Cox proportional hazards modelling. For multivariate analysis, backward modelling was used, with inclusion of variables with *p*-value < 0.1 on univariate analysis. Proportionality of hazards was assessed by visual inspection of Kaplan–Meier curves, and goodness-of-fit assessed using chi-square test on the log ratio. A *p*-value of 0.05 was regarded to be significant.

## Results

The study population comprised 160 patients. Clinicopathological and demographic details are presented in Table 1. 63 cases underwent slide review (39%). In total 11 cases were re-classified on review: 3 cases from PNI positive to negative, 3 cases PNI negative to positive, 2 cases LVI positive to negative and 3 cases cohesive to non-cohesive POI.

**Table 1:** Clinicopathological and demographic features of study population

		Number	%
Sex	Male	101	63.1
	Female	59	36.9
Primary site	Tongue	79	49.4
	Floor of mouth	43	26.9
	Buccal	13	8.1
	Alveolus/RMT/palate	18	11.3
	Lip	7	4.4
Smoking status	Smoker	71	44.4
	Ex-smoker	35	21.9
	Non-smoker	38	23.8
	Unknown	16	10
Alcohol use	Yes	93	58.1
	Ex-drinker	13	8.1
	No	37	23.1
	Unknown	17	10.6
Second head and neck primary	Yes	18	11.3
T-classification (AJCC 8th edition)	T1	67	41.9
	T2	75	46.9
	T3	18	11.3
Depth of invasion	</=5 mm	75	46.9
	> 5 mm, </=10 mm	57	35.6
	> 10 mm	28	17.5
Involved margins (RCPath)	Yes	31	19.4
Involved margins (CAP)	Yes	17	10.6
WPOI-5	Yes	15	9.4
Satellite nodules	Yes	13	8.1
Perineural invasion	Any	34	21.3
	Intratumoural	33	20.6
	Extratumoural	5	3.1
Lymphovascular space invasion	Any	9	5.6
	Intratumoural	8	5.0
	Extratumoural	3	1.9
Postoperative radiotherapy (RT)	No	116	72.5
	RT alone	38	23.8
	Chemoradiotherapy	6	3.8

Fifteen patients (9.4%) were identified as having WPOI-5, of whom 13 (8.1%) had satellite nodules, 5 (3.1%) had extratumoural PNI and 3 (1.9%) had extratumoural LVI. 6 (3.8%) were positive for 2 of the 3 WPOI-5 parameters, but none for all 3.

Mean (median) follow-up for all patients was 61 (55) months (range 1–168 months). 32 patients (20%) developed recurrent disease, including 25 patients with local recurrence (8 concomitant with regional recurrence), 6 with isolated regional recurrence, and 1 with isolated distant recurrence. 26 patients developed second primary cancers, including 18 in the head and neck, of which 12 were in the oral cavity or oropharynx. These patients were censored at the time of presentation with second primary cancers. 58 patients (36.25%) died, of whom 15 (9.4%) died from the index oral cancer, and 43 (26.9%) from other causes.

**Fig. 2:**
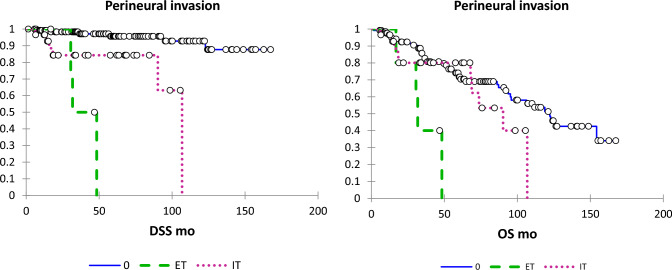
DSS and OS according to PNI (absent, solid line; intratumoural dotted line; extratumoural, dashed line)

**Fig. 3:**
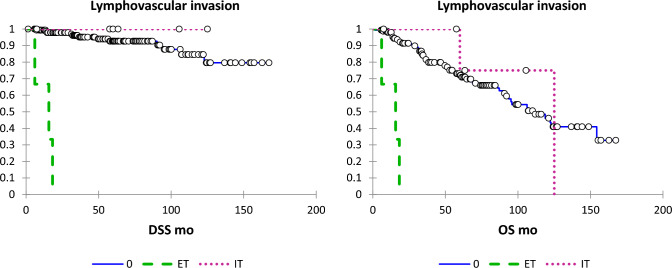
DSS and OS according to LVI (absent, solid line; intratumoural dotted line; extratumoural, dashed line)

**Fig. 4:**
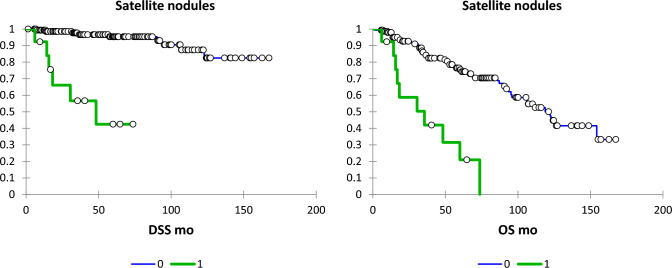
DSS and OS according to satellite nodules (absent, blue line; present, green line)

**Fig. 5:**
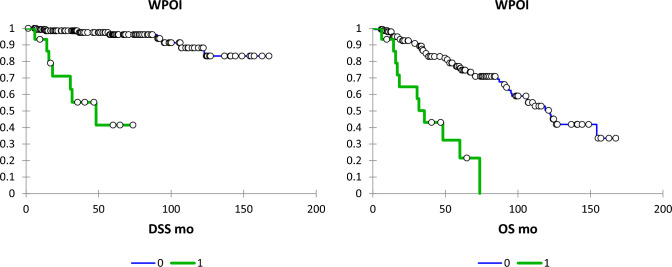
DSS and OS according to WPOI-5 (absent, blue line; present, green line)

Table 2 shows the results of the univariate analysis of clinicopathological factors studied on LRR, disease-specific survival (DSS), and overall survival (OS). WPOI-5 and its 3 constituent components of satellite nodules, extratumoural PNI and extratumoural LVI were all significantly associated with LRR, DSS, and OS. Any PNI was also significantly associated with all of these outcomes. Other factors significantly associated with LRR were non-cohesive POI and intratumoral PNI. For DSS, other significant parameters were DOI > 10 mm, non-cohesive POI, intratumoural PNI, and any LVI. Finally, other parameters which were significant for OS were non-tongue primary tumour site, and postoperative radiotherapy. See Figs. 2, 3, 4, 5 for Kaplan–Meier survival curves for PNI, LVI, satellite nodules and WPOI-5, respectively.

**Table 2:** Univariate analysis of impact of pathological features and postoperative radiotherapy on DSS and OS

	LRR OR (95% CI)	LRR p-value	DSS OR (95% CI)	DSS p-value	OS OR (95% CI)	OS p-value
Non-tongue primary site (vs tongue)	0.75 (0.36, 1.53)	0.43	0.92 (0.33, 2.54)	0.78	2.01 (1,17–3.47)	0.01
> 5 mm depth of invasion	1.60 (0.75, 3.40)	0.23	2.26 (0.72–7.09)	0.16	0.91 (0.54–1.53)	0.71
> 10 mm depth of invasion	1.52 (0.65, 3.55)	0.33	3.04 (1.03–8.97)	0.04	1.18 (0.58–2.41)	0.65
> 20 mm diameter	1.00 (0.45, 2.24)	0.99	2.14 (0.76–6.05)	0.15	1.27 (0.71–2.26)	0.42
Non-cohesive pattern of invasion	2.09 (1.02, 4.30)	0.04	4.22 (1.48–12.09)	0.007	1.62 (0.94–2.79)	0.08
Involved margins (RCPath)	0.84 (0.34, 2.07)	0.71	0.80 (0.22–2.87)	0.73	0.50 (0.23–1.06)	0.07
Involved margins (CAP)	0.98 (0.34, 2.84)	0.97	0.46 (0.06–3.56)	0.46	0.49 (0.18–1.36)	0.17
Any PNI	4.46 (2.14, 9.30)	< 0.0001	10.23 (3.29–31.84)	< 0.0001	1.97 (1.06–3.66)	0.03
Extratumoral PNI	8.01 (2.30, 27.83)	0.001	14.46 (3.69–56.70)	0.0001	5.30 (1.84–15.22)	0.002
Intratumoral PNI	3.96 (1.89, 8.27)	0.0003	7.62 (2.55–22.75)	0.0001	1.81 (0.96–3.43)	0.07
Any LVI	2.09 (0.63, 6.91)	0.23	4.73 (1.33–16.79)	0.02	1.88 (0.75–4.72)	0.18
Extratumoral LVI	23.46 (6.35, 86.62)	< 0.0001	73.50 (14.61–369.80)	< 0.0001	22.65 (6.17–83.1)	< 0.0001
Intratumoral LVI	1.36 (0.32, 5.74)	0.67	2.93 (0.66–13.01)	0.16	1.48 (0.54–4.10)	0.45
Satellite nodules	6.53 (2.71, 15.72)	< 0.0001	18.32 (5.53–60.67)	< 0.0001	5.45 (2.66–11.15)	< 0.0001
WPOI-5	6.77 (2.89, 15.84)	< 0.0001	22.68 (6.54–78.68)	< 0.0001	5.31 (2.64–10.65)	< 0.0001
Postoperative radiotherapy	1.05 (0.49, 2.26)	0.90	0.37 (0.08–1.65)	0.19	0.41 (0.20–0.85)	0.02

Table 3 gives the results of the multivariate analysis for OS. Variables removed from the model are not presented. Satellite nodules (odds ratio 6.61, 95% CI 2.83–15.44, *p* < 0.001) and extratumoural LVI (odds ratio 9.97, 95% CI 2.19–45.35, *p* = 0003) were independently associated with OS. In addition, significant associations were found for post-operative radiotherapy (odds ratio 0.40, 95% CI 0.19–0.87, *p* = 0.02) and non-tongue subsite (odds ratio 3.03, 95% CI 1.70–5.39, *p* = 0.0002). For LRR, significant factors on multivariate analysis were extratumoural LVI (odds ratio 13.49, 95% CI 3.19–56.99); extratumoural PNI (odds ratio 4.65, 95% CI 1.17–18.54), and any PNI (odds ratio 2.92, 95% CI 1.23–6.93) (Table 4). DSS was not assessed as part of the multivariate analysis due to the small number of events. Finally, due to interaction between WPOI and component variables of this metric, a further multivariate analysis for OS was performed including primary site, postoperative radiotherapy, RCPath margins, and WPOI-5. This analysis demonstrated a strong impact of WPOI-5 on OS (odds ratio 7.57, 95% CI 3.67–15.60). Non-tongue subsite (odds ratio 2.82, 95% CI 1.61–4.95) and postoperative radiotherapy (odds ratio 0.41, 95% CI 0.19–0.88) were once again also significant.

**Table 3:** Multivariate analysis of impact of pathological features and postoperative radiotherapy on OS

	OS OR (95% CI)	OS p-value
Extratumoral LVI	9.97 (2.19–45.35)	0.003
Satellite nodules	6.61 (2.83–15.44)	< 0.0001
Margins (RCPath)	2.06 (0.93–4.53)	0.07
Postoperative radiotherapy	0.40 (0.19–0.87)	0.02
Tumour subsite non-tongue	3.03 (1.70, 5.39)	0.0002

**Table 4:** Multivariate analysis of impact of pathological features on LRR

Extratumoral LVI	13.49 (3.13, 56.99)	0.0004
Any PNI	2.92 (1.23, 6.93)	0.02
Extratumoral PNI	4.65 (1.17, 18.54)	0.03

## Discussion

Depth of invasion was incorporated into the T staging of oral cavity SCC in TNM8 to enable clinicians to more accurately discriminate higher risk early-stage cancers, which may benefit from adjuvant radiotherapy [1]. However, there is still an imperative to identify additional adverse prognosticators in stage I/II disease. Worst pattern of invasion at the invasive tumour front is one such potential high-risk feature. WPOI has been found in multiple studies to predict for poor outcome in OSCC, with WPOI-5 versus patterns 1–4 representing the key significant cut-point [19, 21, 32–34]. WPOI-5 is defined as tumour dispersion of ≥ 1 mm between satellite foci and includes extratumoural PNI and LVI. Of note, WPOI has previously been validated as a prognosticator specifically in early-stage OCSCC. Li et al. found WPOI-5 to significantly correlate with both locoregional recurrence and disease-specific survival on multivariate analysis in a stage I/II cohort. When WPOI-5, was present the probability of developing locoregional recurrence was 42% [21]. We also found WPOI-5 to be significantly associated with survival on univariate analysis. In our multivariate model, where we included WPOI-5, as well as its components, the subcategories of satellite nodules and extratumoural LVI were found to be independently predictive of OS, whereas overall WPOI-5 was not significant.

Interestingly the RCPath and CAP differ in their current recommended approaches to reporting of POI. The RCPath advocates mandatory use of a 2-tier system of cohesive versus non-cohesive with the latter including patterns 4 and 5 [35]. The RCPath protocol is currently under review and it remains to be seen if this cut-point is modified in the updated guidelines, given that based on published data WPOI-5 is most significant [19, 21, 32–34]. In contrast the current CAP protocol categorises WPOI-5 as an optional data element due to overlap with the reporting of extratumoural PNI and LVI [36]. However, this approach overlooks the potential importance of satellite nodules as an independent and standalone prognostic factor. Of note, in our cohort, of the 15 patients fulfilling WPOI-5 criteria a majority (13 patients) had satellites nodules, of whom 7 had this criterion alone.

Perineural invasion is a well-recognized adverse prognosticator in OCSCC including in early-stage node negative cases. It has been shown to correlate with locoregional recurrence, nodal metastatic spread and reduced disease-specific and overall survival [17–25]. Furthermore, the National Comprehensive Cancer Network (NCCN) recommends adjuvant radiotherapy for surgically resected T1/T2N0 oral cavity cancers with adverse risk features such as PNI [39]. An additional emerging data element is the location of PNI. Miller et al. reported a trend toward reduced disease-free survival (DFS) with extratumoural PNI [40]. More recently, Park et al. demonstrated worse DFS on multivariate analysis in cases with this feature [41]. On univariate analysis, we demonstrated a significant correlation between all categories of PNI and reduced DSS. Any PNI and extratumoural PNI also predicted for worse OS. However, all 3 PNI groupings lost significance on multivariate analysis. This may be secondary to the impact of postoperative radiotherapy (PORT), or interaction with other factors such as satellite nodules and lymphovascular invasion in the multivariate model.

Lymphovascular space invasion (LVI) has been linked to an increased risk of nodal metastases, locoregional recurrence and reduced survival outcomes in OCSCC [22, 24, 26–31]. Similar to PNI it is listed by the NCCN as an indication for postoperative radiotherapy in early-stage disease [39]. A recently published meta-analysis investigating the impact of LVI in OCSCC demonstrated a significant association between presence of LVI and reduced DSS and OS. In addition, a positive correlation between cervical nodal metastasis and LVI was found in low-stage disease and therefore it could be used as a criterion to select patients for neck dissection [30]. We found extratumoural LVI, but not intratumoural LVI to significantly predict for OS on multivariate analysis.

The main limitations of this study are its retrospective nature, and the small number of patients dying from cancer precluding multivariate analysis of disease-specific survival. A further limitation is inclusion of TNM8 T3 cases, but this is mitigated by inclusion of DOI as a variable. Furthermore, DOI measurements were previously re-evaluated for all patients in our study cohort who underwent surgery prior to the publication by the AJCC of the clarified DOI definition in 2017. Finally, while WPOI-5 and its component parameters showed very strong significance on univariate analysis, caution should be exercised in interpreting the results of the multivariate analysis, due to the large number of variables entered in the model, the potential for interaction between the variables, and the small number of events. On the other hand, strengths include consensus slide review by 2 pathologists to assess for WPOI-5 criteria and re-categorisation of PNI and LVI status of cases if appropriate. It may be viewed as an additional limitation that not all cases underwent slide review. However, this would not overcome the issue of both interobserver and intraobserver variability in the evaluation of WPOI-5 parameters. In a prior study of floor of mouth SCC, we found interobserver agreement to be substantial for LVI, but only moderate for POI [42]. Interobserver agreement for the assessment of PNI in OCSCC has been shown to range from fair to at best moderate [43, 44].

## Conclusion

WPOI-5 predicts survival in early oral cancer, with the subcategories of satellite nodules and extratumoural LVI independent prognosticators of OS in our cohort, along with PORT. Our results support the NCCN recommendation that adjuvant therapy be considered for surgically resected T1/T2N0 oral cavity cancers with PNI and/or LVI [39] and suggest that satellite nodules should also be regarded as an adverse risk factor in this patient cohort. Further study is recommended to assess the benefits of adjuvant treatment for patients with satellite nodules and to ascertain if reporting of satellite nodules independent of other WPOI-5 parameters should be a required data element in both RCPath and CAP guidelines.
